# Performance in Physical Education and Health Impairment 30 Years Later—A Community Based Cohort Study

**DOI:** 10.1371/journal.pone.0035718

**Published:** 2012-04-23

**Authors:** Simon Timpka, Ingemar F. Petersson, Rebecca Rylance, Ljuba Kedza, Martin Englund

**Affiliations:** 1 Musculoskeletal Sciences, Department of Orthopedics, Clinical Sciences Lund, Lund University, Lund, Sweden; 2 Clinical Epidemiology Research & Training Unit, Boston University School of Medicine, Boston, Massachusetts, United States of America; Tehran University of Medical Sciences, Iran (Islamic Republic Of)

## Abstract

**Objective:**

A main purpose of physical education (PE) in school is to promote future health. However, there is very limited evidence of the effects of PE on the adult health. We hypothesized that a low performance in PE was associated with an increased risk of health impairment by middle age.

**Methods:**

We performed a cohort study in a community-based setting in Sweden spanning over three decades. We followed up on 1712 of 2225 students (76.9%) who in 1974–1976 graduated with a grade in PE after 9 years of education (mean subject age 16 years). The grade in PE (compulsory subject) was retrieved from municipal archives. We defined three proxies for health impairment: total number of visits to primary care physicians in 2003–2007, having been hospitalized 2003–2007, and total number of days with sick leave in 2004–2007. Using binomial regression models, we adjusted the risk estimates for level of education and occupation. Subjects with an average grade in PE served as reference category.

**Results:**

In both the crude and adjusted model, women with a low grade in PE had more physician visits (adjusted IRR 1.30, 95% confidence interval 1.06–1.60) and an increased number of days with sick leave (adjusted IRR 1.44, 1.05–1.95). An increased, although not significant, risk was also observed for having received in-patient care (adjusted RR 1.26; 0.88–1.80). No significant results or similar pattern were observed in men.

**Conclusion:**

Women with a low grade in PE in adolescence seem to have an increased risk of health impairment by middle age, raising the question of early primary prevention towards these students in particular.

## Introduction

Primary prevention of disease is becoming increasingly important in an aging population. A common form of early primary prevention is physical education (PE). The recurring reports of prevalent child obesity [1]–[3] and physical inactivity [4], [5] have put focus on the more immediate positive effects of physical activity on the health of children [6]–[8]. Furthermore, interventions of increased or modified PE in the school setting have shown to reduce risk factors for future disease [9], [10]. It is also believed that PE mediates a carry-over effect by engaging students in sports and physical activity throughout childhood and adolescence [11], [12]. However, although this expected carry-over effect remains a cornerstone of the subject's theoretical framework [11], there is very limited evidence of long-term effects of PE on actual health impairment by middle-age or older adult life, probably due to the very long follow-up required [13].

In Sweden, PE in the compulsory school took its more modern form in the 1960s. However, although uncommon in Sweden today, girls and boys were in the 1970s still recommended to be taught separately [14]. A main goal of the subject was to raise awareness of the link between physical activity and health. For students aged 13–16 years, the allocated time of PE in the curriculum was three hourly lessons per week. Although preadolescent students of both sexes were in general considered to benefit from the same types of physical activity, by puberty it was recommended that the teacher adapt the lessons to better accommodate an assumed sex-specific need of activity. However, a main goal of PE was to ensure that each student would have the opportunity to try various forms of physical activity; it was only the main focus that should differ between the sexes. Each student should have had the opportunity to try each recommended activity every second semester. These activities included, but were not strictly limited to the following; gymnastics, dancing, ball sports, athletics, and orienteering, skating, skiing, and swimming. In summary, teenage girls were recommended more esthetic features and gymnastics while the education for boys were more focused on ball sports and strength.

The aim of this community-based cohort study, using register linkage of personal data, was to investigate the association between the grade in PE actually received in school and health impairment 30 years later. We used three different proxies for health impairment (our outcome): 1) number of visits to physicians in primary out-patient care, 2) having been hospitalized, and 3) number of days with sick leave. We hypothesized students with a low grade in PE to be at increased risk of health impairment by middle age.

## Methods

For this longitudinal study we used historical exposure and outcome data. Importantly, both the exposure information and the two outcomes were prospectively registered in the resources that we retrieved the data from; municipal archives of school grades, the Skåne Health Care Register [15], [16], and the Swedish Social Insurance Agency [17]. We prepared the manuscript according to the STROBE-checklist [18].

### Ethics statement

The study was conducted according to the Declaration of Helsinki and approved by the Ethical Review Board of Lund University, Sweden. Only register-data made anonymous was used for analyzes. The individuals in the cohort were informed of the study and offered “opt-out” via regional news press, a process sanctioned by the Ethical Review Board.

### Study sample

We included all students (typically aged 16 years) who in 1974 to 1976 had completed nine years of compulsory education in the municipality of Lund (total population in 1975: 76,284) in southern Sweden. We identified a total of 2335 graduates from the hand-written municipal archive of whom 2298 (98.4%) had a complete and valid 10-digit personal identification number.

### Collection and linkage of data

The grade in PE (5-item categorical scale where 1 = the worst grade and 5 = the best grade) were retrieved manually from municipal records along with the personal identification number. At the time of these students' schooling, a relative grade based on a theoretical normal distribution was used. The students were thus measured by their relative abilities (i.e. theoretically to the whole body of students in the same grade on a national level) and not by their absolute abilities. A similar rational has since been applied to the European Credit Transfer and Accumulation System (ECTS) of the European Union [19]. The percentage of students given each grade was ideally 7-24-38-24-7 (grade 1–5), but this was allowed to differ locally depending on the characteristics of the students in each class. Boys and girls were taught separately by a same sex teacher.

We ensured via the national population register that subjects were still alive and residing in the county of Skåne from 2003 to 2007. We then linked our data to a regional register covering both in-patient health care and primary care visits, of the population of the southernmost county of Sweden.

We retrieved all data on sick leave for the period 2004 to 2007 from the Swedish Social Insurance Agency. Excluding the first day of absenteeism, for which no compensation is given, the employer pays the first two weeks of an employee's sick leave. Hence, sick leave of short duration for employees (≤14 days), is not included in the data. However, students, self-employed, and unemployed are included from day two. A certificate from a physician is mandatory for all from day eight.

Furthermore, we also retrieved data from Statistics Sweden including income 2006, level of education achieved, and occupation according to the Swedish three digit version of International standard classification of occupations (ISCO-88) [20].

Importantly, data from all the five register resources (municipal archives of school grades, national population register, Skåne Health Care Register, the Swedish Social Insurance Agency, and Statistics Sweden) were all linked based on each subject's unique personal identification number.

### Outcome variables

Using the regional health care register 2003–2007, we identified all visits to physician in primary health care and hospitalizations. We also collected the number of days with sick leave compensation 2004–2007 from the Swedish Social Insurance Agency.

### Statistical analysis

All statistical analyses were calculated with STATA software 11.0 (Copyright 1984–2009 StataCorp). To increase study power, we combined the two lower PE grades and the two higher PE grades to get a total of three groups; low (1 and 2), average (3), and high (4 and 5) PE grade.

For physician visits in primary care and number of days with sick leave, we used a negative binomial regression model to calculate unadjusted sex-stratified incidence rate ratios (IRR), with 95% confidence interval (95% CI). For in-patient care we instead used a dichotomous outcome (hospitalized/not hospitalized) in a logistic binomial model to estimate relative risks (RRs). A PE grade of 3 served as the reference category.

We adjusted the models for education and occupation (considered to be the primary model). The level of education was divided into four groups (9 years or less, 10–12 years, 13–15 years, 16+ years). Occupation was also divided into four groups using a blue collar/white collar approach based on the major groups of the ISCO-88 classification.

Furthermore, we investigated the confounding effect of general academic performance in adolescence on the adjusted model. In the subjects Mathematics and English, each student took either the general or the special course. In Mathematics, the special course made students eligible for further education in the natural sciences. In English however, the two courses mainly differed in tempo during lessons. We grouped academic performance into three categories; low (those who either had not taken any special course or received a grade lower than 2 when doing so), average (those who had received a grade of at least 2 in one of the special courses), and high (those who had received a grade of at least 2 in both courses).

To investigate any potential bias introduced by employment status on the risk of sick leave, we performed a sensitivity analysis, in which individuals with no formal employment in 2006 were excluded.

Further, we used data on income, education, and occupation from Statistics Sweden to characterize the loss to follow-up (typically because of relocation outside the county).

## Results

**Figure 1:**
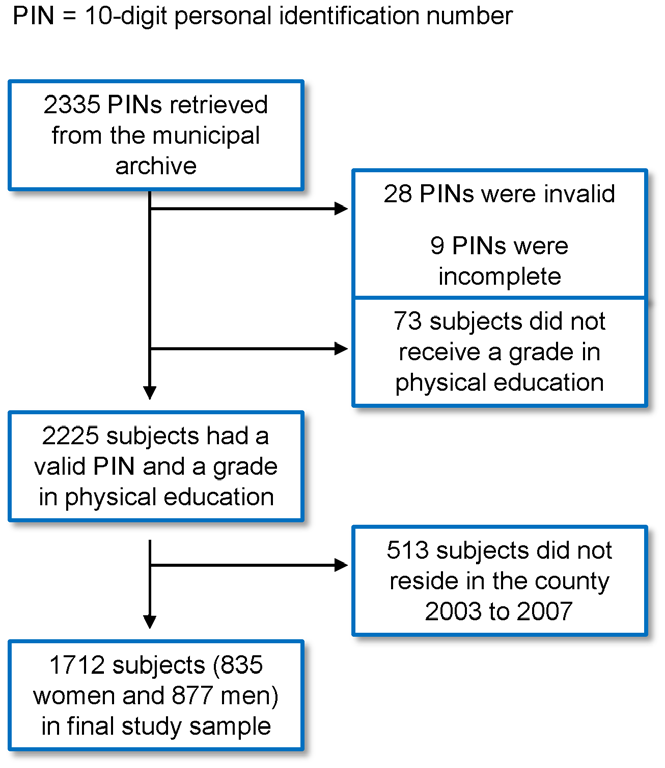
Flowchart detailing the identification of the study sample and loss to follow-up.

Out of 2335 personal identification numbers retrieved from the handwritten municipal archives, we confirmed 2225 subjects having a valid personal identification number and a valid grade in PE. Of those, 1712 (76.9%) remained alive and resident in the county during the follow-up period (Figure 1). The subjects were born 1957 to 1962 with 97.5% born 1958 to 1960. Their mean (SD) age by the end of the study period (Dec 31^st^ 2007) was 48.0 (1.4) years and 48.8% were women. Subjects lost to follow-up had a greater proportion with high grade in PE, higher median income, and a higher level of education (Table 1).

**Table 1:** Descriptive statistics of the study sample and loss to follow-up.

		Women		Men	
		Study sample	Loss to follow-up	Study Sample	Loss to follow-up
Number		835	241 (22.4%)	877	272 (23.7%)
Calculated age by Dec 31^st^ 2007, mean (SD)		48.0±0.9	48.0±0.9	48.0±0.9	48.0±0.9
**Physical education grade (%)**					
	Low	22.3	16.2	28.7	22.1
	Average	39.9	35.2	36.6	39.3
	High	37.8	48.6	34.7	38.6
**Level of education (%)**					
	9 years or less	10.8	2.5	17.2	3.7
	10–12 years	40.4	17.0	46.3	19.5
	13–15 years	35.8	30.3	23.7	21.0
	16+ years	13.0	24.9	12.8	27.6
	Not registered (i.e. at least 9 years)	0	25.3	0	28.3
**Occupation** a **(%)**					
	Group 1	4.2	4.2	10.4	12.1
	Group 2	45.0	49.0	39.3	40.1
	Group 3	34.1	11.6	10.9	4.8
	Group 4	9.3	2.9	29.1	6.6
	Not registered	7.3	32.4	10.3	36.4
**Income 2006 (Euro)** b					
	Median	23 447	28 424	31 421	37 461
	25 percentile	13 593	15 698	20 253	13 774
	75 percentile	29 921	40 116	41 631	58 979

a = Divided according to ISCO-88: Legislators, senior officials and managers” in group 1, “Professionals” and “Technicians and associate professionals” in group 2, “Clerks” and “Service workers and shop and market sales workers” in group 3, with the remaining ISCO-88 major groups, consisting of blue collar workers and military personal, in group 4.

b = Missing values (n = 146) excluded from loss to follow-up.

Adjusted for education and occupation, women with a low grade in PE had more physician visits in primary care as well as more days with sick leave (Table 2). A similar, although not significant, risk increase was also observed for in-patient care. However, no correspondent pattern was observed in men. High grade in PE in men and women was neither associated with increased nor decreased risk for health impairment.

**Table 2:** Risk estimates for proxies of health impairment by grade in physical education (PE).

Outcome	Women				Men			
	Low PE Grade		High PE grade		Low PE Grade		High PE grade	
	Crude IRR^a^ (95% CI)	Adjusted IRR^a^^, ^ b (95% CI)	Crude IRR^a^ (95% CI)	Adjusted IRR^a^^, ^ b (95% CI)	Crude IRR^a^ (95% CI)	Adjusted IRR^a^^, ^ b (95% CI)	Crude IRR^a^ (95% CI)	Adjusted IRR^a^^, ^ b (95% CI)
Visits to GPs^c^	**1.42** (1.17; 1.72)	**1.30** (1.06; 1.60)	1.01 (0.85; 1.20)	1.06 (0.89; 1.26)	1.18 (0.97; 1.42)	1.14 (0.93; 1.40)	0.93 (0.77; 1.11)	0.90 (0.75; 1.09)
Sick leave	**1.37** (1.01; 1.84)	**1.44** (1.05; 1.95)	0.84 (0.64; 1.12)	0.86 (0.64; 1.15)	1.34 (0.95; 1.90)	1.06 (0.73; 1.53)	1.05 (0.74; 1.48)	1.06 (0.73; 1.52)
	Crude RR^a^ (95% CI)	Adjusted RR^a^^, ^ b (95% CI)	Crude RR^a^ (95% CI)	Adjusted RR^a^^, ^ b (95% CI)	Crude RR^a^ (95% CI)	Adjusted RR^a^^, ^ b (95% CI)	Crude RR^a^ (95% CI)	Adjusted RR^a^^, ^ b (95% CI)
Hospitalization	1.18 (0.85; 1.65)	1.26 (0.88; 1.80)	0.82 (0.59; 1.14)	0.79 (0.55; 1.12)	0.91 (0.61; 1.34)	0.70 (0.44; 1.10)	1.10 (0.78; 1.55)	1.11 (0.76; 1.61)

Significant point estimates in bold.

95% CI = 95% confidence interval.

IRR = Incidence rate ratio.

RR = Relative risk.

a = Compared to an average grade.

b = Adjusted for education and occupation.

c = i.e. visits to physicians in primary health care.

The sensitivity analysis of academic performance resulted only in minor risk adjustments towards 1. The largest risk difference was observed for number of days with sick leave in women with a low PE grade (sensitivity IRR 1.37, 1.00–1.87 vs. adjusted IRR 1.44, 1.05–1.95).

The sensitivity analysis for risk of sick leave (excluding 195 individuals) showed very little effect of employment status, leaving IRRs, for all groups but women with a low PE grade, unaffected or adjusted towards 1 (data not shown). The difference was small also in women with a low PE grade. However, the association was strengthened somewhat (IRR 1.51, 1.09–2.09) compared to the primary model.

## Discussion

To investigate the association between the performance in PE in youth and health impairment 30 years later, we used number of visits to physicians in primary health care, having been hospitalized, and number of days with sick leave as proxies for health impairment. We found that women with a low grade in PE had more days with sick leave and more physician visits in primary care than women with an average grade. The findings suggest that women with a low PE grade have an increased risk of health impairment when they reach middle age, the group thus being an important target for primary prevention efforts.

### Possible explanations for associations and gender aspects

We have earlier postulated that the increased risk of musculoskeletal disease in women with a low PE grade can be explained by a bio-psycho-social model [21]. We propose that a similar model might also explain the associations observed in the present study. Though diseases can differ in etiology and clinical presentation, many diagnoses, as well as sick leave [22], share common risk factors such as smoking [23]–[25], obesity [26]–[28], and inadequate levels of physical activity [29]. Supporting our model, the clustering of risk factors of disease, such as smoking and diet, has been reported in both adolescents and adults [30], [31]. Furthermore, smoking is in young women associated with a being physically inactive while it is inversely associated with a high fiber diet [32]. In the US, cigarette use is also associated with a decline in physical activity during adolescence in white girls [33]. Studies have also shown that a low performance in PE is associated with an increased risk of later schizophrenia [34], [35]. Furthermore, our model is supported by two studies with baseline data from the same time period [36], [37]. Using grades based on the same curriculum as the grades in the present study, the first study reports a reduced risk of smoking in adulthood in girls with a high grade in PE [36]. Furthermore, a high grade was associated with increased cardiovascular fitness in both boys and girls, whereas only boys with a high grade had increased levels of physical activity. These results are somewhat supported by a study reporting the PE grade in adolescence to be a predictor of later physical activity for boys but not for girls [12]. Out of a set of variables collected in adolescence, the second study identified the PE grade as the strongest predictor of exercise in the middle age [37].

In contrast to their female counterparts, men with a low grade in PE did not appear to have increased risk for future health impairment. Explanatory factors at both baseline and follow-up might be influenced by sex or gender. In students with a low PE grade, it is feasible that the reported gender differences in structural and behavioral determinants of health [38], as well as health care utilization [39], might mediate part of the gender difference in risk of future health impairment. However, it has also been reported that girls have benefited from PE interventions in form of better fitness [40], increased adult levels of physical activity [10], and decreased sedentary behavior [41] when boys have not. This could potentially be explained by boys being more physically active in general [42] and less anxious of PE [43]. With the habitual physical activity level of boys being higher than that of girls, an absolute increase in physical activity might affect boys' health less than girls'. Furthermore, the PE curriculum was at the time of the subjects' education somewhat depending on sex. Thus, the PE grade as such might reflect different abilities in girls as compared to boys, .e.g. dancing being more important for grading in girls whereas focusing less on ball sports. On the other hand, we cannot exclude the possibility that there is a third variable explaining the difference between boys and girls, being associated with both PE grade and later health impairment. For instance, in terms of not getting a low grade, self-esteem could be a more important factor in girls than in boys.

### Adjustment of confounders and the concept of health impairment

The aim of this study was not to present a causal explanation, but merely to investigate the association between PE grade and later health impairment in the middle age. Occupation and level of education are known risk factors [22] for sickness absence, both being a better determinant than income [44]. Socioeconomic status is also associated with health, the divergence partly mediated through individual risk factors [45]. Therefore, to facilitate interpretation, we adjusted the risk estimates for educational length and occupation, and the effect of adjustment was only modest. Primarily, we did not consider other school grades as confounders because our hypothesis was based on the actual grade in PE and not the grade as a proxy for e.g. physical fitness or physical activity. However, we observed only minor adjustments of the risk estimates when we included academic performance in an adjusted sensitivity analyses.

For the purpose of this study, we consider hospitalization and the number of visits to a physician in primary health care in combination with sick leave data to be acceptable proxies for health impairment. The comprehensive regional health care register is the basis for compensating health care providers for their service. As the health care system in Sweden is publicly financed all health care providers, with few exemptions, are included in the register. Moreover, the sick leave register is based on payments made to individuals by the Swedish Social Insurance Agency. All in all, we view the registers as a reliable source of outcome for the present study. In terms of health impairment, we regard primary health care visits and hospitalization to be somewhat complimentary; the former being a better proxy for illness, the latter being more focused on disease. Physician visits are associated with self-perceived health in both the adult [46] and working-age population [47], in the latter showing a linear trend. Likewise, spells of sickness absence are associated with poor self-perceived health [48], [49] and increased risk of mortality [50], [51]. Moreover, far from all visits to a physician result in sick leave.

### Limitations

There are important limitations that we would like to acknowledge: First and most importantly, we lack information on students' smoking, anthropometry, and physical activity which all are indicators for future health. In fact, these covariates may act as either confounders or intermediate variables on a causal chain (from physical fitness/activity/sporting ability in school to health as an adult). Even so, we were interested in the predictive capabilities of the grade received in PE as such. Second, the subjects lost to follow-up differed from the rest of the cohort by having higher PE grade, higher level of education and higher income. The differences are probably attributable to higher education being associated with more frequent relocation. Most likely the subjects who have relocated have on average better health, due to the higher socioeconomic status [45], which if true will bias our results into finding decreased risks if having low PE grade, and increased risks if having high PE grade, further strengthening our findings. Third, the day of inclusion in the sick leave register depends on employment status. However, when we performed a sensitivity analysis, excluding all individuals without formal employment from the model, the results appeared essentially the same. Fourth, the municipality under study includes a university town with inhabitants that might differ from the general population. However, simultaneously with the start of the study, adjacent rural municipalities were incorporated in the study municipality, thus possibly lessening this effect. Nevertheless, the study's regional approach calls for larger studies. Fifth and finally, we have not controlled for possible pre-existing disease at baseline. However, we have only included subjects with a valid grade in PE, thus excluding those students who were exempt from PE because of medical reasons.

### Conclusions

In summary, our study suggests that women with a low performance in PE in adolescence have increased risk of health impairment by middle age. In men, the data did not support a similar conclusion. We speculate that this gender difference might partly be explained by different physical activity patterns in youth, as well as differing determinants of health in adulthood. Our findings raise the question of directed primary prevention towards girls with low performance in PE. Further studies are needed to better identify the strength of this association and its cause and effect relationship. At last, we would like to encourage researchers in the field to consider gender aspects when planning and presenting future studies, especially enabling stratified analysis on gender in future RCTs.
